# Acoustic Noise Alters Selective Attention Processes as Indicated by Direct Current (DC) Brain Potential Changes

**DOI:** 10.3390/ijerph111009938

**Published:** 2014-09-26

**Authors:** Karin Trimmel, Julia Schätzer, Michael Trimmel

**Affiliations:** 1 Department of Neurology, Medical University of Vienna, Vienna 1090, Austria; E-Mail: karin.trimmel@meduniwien.ac.at; 2 Department of Psychology, University of Vienna, Vienna 1010, Austria; E-Mail: julia.schaetzer@gmail.com; 3 Institute for Environmental Hygiene, Center for Public Health, Medical University of Vienna, Vienna 1090, Austria

**Keywords:** acoustic noise, facilitation and inhibition, direction of attention, brain DC potentials, attention control, attention shift

## Abstract

Acoustic environmental noise, even of low to moderate intensity, is known to adversely affect information processing in animals and humans via attention mechanisms. In particular, facilitation and inhibition of information processing are basic functions of selective attention. Such mechanisms can be investigated by analyzing brain potentials under conditions of externally directed attention (intake of environmental information) *versus* internally directed attention (rejection of environmental stimuli and focusing on memory/planning processes). This study investigated brain direct current (DC) potential shifts—which are discussed to represent different states of cortical activation—of tasks that require intake and rejection of environmental information under noise. It was hypothesized that without background noise rejection tasks would show more positive DC potential changes compared to intake tasks and that under noise both kinds of tasks would show positive DC shifts as an expression of cortical inhibition caused by noise. DC potential shifts during intake and rejection tasks were analyzed at 16 standard locations in 45 persons during irrelevant speech or white noise *vs.* control condition. Without noise, rejection tasks were associated with more positive DC potential changes compared to intake tasks. During background noise, however, this difference disappeared and both kinds of tasks led to positive DC shifts. Results suggest—besides some limitations—that noise modulates selective attention mechanisms by switching to an environmental information processing and noise rejection mode, which could represent a suggested “attention shift”. Implications for fMRI studies as well as for public health in learning and performance environments including susceptible persons are discussed.

## 1. Introduction

Environmental acoustic noise (even of low to moderate intensity) is known to have—besides health effects [1,2,3,4,5,6,7]—a number of adverse effects on human information processing by modulating attention [8,9,10,11]. The function of attention has already been described by James [12] “Every one [sic] knows what attention is. It is the taking possession by the mind, in clear and vivid form, of one out of what seem several simultaneously possible objects or trains of thought. Focalization, concentration, [sic] of consciousness are of its essence. It implies withdrawal of some things in order to deal effectively with others, and is a condition which has a real opposite in the confused, dazed, scatter-brained state, which in French is called *distraction*, and *Zerstreutheit* in German” pp. 403–404). In attention processes, facilitation of relevant information and inhibition of non-relevant stimuli are seen as basic underlying mechanisms of selective attention [13]. This means that during complex cognitive tasks, non-task-relevant information is filtered out as an expression of selective attention. Rejection of environmental stimuli would therefore take place in tasks that require attention to be directed towards internal processing or in cases where environmental stimuli would affect current information processing. Acoustic noise has often been investigated as it is part of everyday life and is therefore of high practical relevance. The present study aims to investigate the effect of acoustic background noise on an aspect of a neurophysiological indication of brain processes, namely cortical direct current (DC) potentials, which have been discussed to reflect different states of cortical activation [14,15,16].

In the present study, effects of facilitation and inhibition processes of selective attention were investigated by manipulating the direction of attention towards environmental stimuli or towards internal mental activity. According to Lacey [17], attention tasks that require observation of environmental stimuli are referred to as “environmental intake” tasks and attention tasks that require attention to be directed towards internal processing are labeled “environmental rejection” tasks. Neurophysiological correlates of the direction of attention have been investigated with EEG recordings [18], reporting more alpha activity during internally directed attention tasks compared to externally directed attention tasks. Additionally, higher alpha amplitudes have been observed during internally directed attention tasks [19,20] which was interpreted as an expression of inhibition of non-task-relevant information. However, currently no data exist to examine whether direction of attention modulates brain DC potential shifts, and whether this interacts with acoustic environmental background noise, which poses the topic of the present study.

The origins of DC potentials and their shifts are–besides some contribution by glial cells [21,22]—considered to be mainly neuronal representing hypo- and hyperpolarization of pyramidal cells at their apical dendrites with underlying complex interactions between the cortex, thalamus, reticular formation, and the basal ganglia [21,22,23]. DC potential shifts may also be altered by changes in partial pressure of carbon dioxide (pCO_2_) [24] and therefore by respiration rates [25,26]. However, recent fMRI findings [27] support the view that changes in DC potentials by investigating infra-slow fluctuations (ISFs) in scalp potentials reflect changes in cortical excitability and still have functional significance on the execution of cognitive tasks [28,29].

It has been discussed that negative DC potential shifts recorded from the scalp [15,30,31] as well as from single cell recordings [32] are a sign of higher excitability and therefore reflect cortical activation, which is associated for instance with better performance and shorter reaction times [14,30]. In addition, there is also some evidence that facilitation of processing of environmental information is associated with preceding negative DC potential shifts [31] and moreover, higher *sustained* negative DC potentials were found in association with shorter reaction times [33]. 

In contrast, positive DC potential shifts, which might occur during sleep [34], but also during processes of selective attention, are seen as a sign of reduced excitability or cortical inhibition [15,35,36]. In regard to noise, Trimmel and Poelzl [9] found more frontal positivity and less parietal negativity for a spatial-cueing paradigm [37] under acoustical noise conditions (*i.e.*, a mixture of environmental low intensity sounds) compared to no-noise conditions, which was interpreted as an expression of filtering out irrelevant information.

Therefore, in the present study it was hypothesized that brain DC potentials differ for intake and rejection tasks and that rejection tasks are accompanied by more positive DC potential shifts compared to intake tasks. It was furthermore expected that under noise conditions, intake tasks would also be associated with more positive DC potentials compared to no-noise conditions as an expression of rejection of background noise. In order to take into account modality effects [38,39], intake and rejection tasks were both investigated by verbal and visual figural tasks. Noise effects were investigated by both irrelevant speech and white noise to give some indication of the generality of noise effects.

## 2. Material and Methods

### 2.1. Participants

Forty-eight participants (30 females) with an age range of 19–37 years (mean age and SD 24 ± 5 years) participated in the study. Participation was voluntary (for course credit) and all persons were right-handed, non-medicated and had normal or corrected-to-normal vision. The experiment was ethically and formally approved by the University of Vienna and informed consent was obtained from the participants.

### 2.2. Design

The study was based on a 2 (Noise Type; irrelevant speech *vs.* white noise) × 2 (Noise Condition; noise *vs.* no-noise) × 2 (Direction of Attention; intake *vs.* rejection) × 2 (Modality of attended stimuli; figural *vs.* verbal) × 16 (Recording Location) ANOVA design with the last four factors as repeated measures. The sequences of the four attention tasks (intake-figural, intake-verbal, rejection-figural, rejection-verbal) as well as of noise condition were balanced across participants. The sequence of the attention tasks was kept constant within persons for the noise and the no-noise condition.

### 2.3. DC Recordings

DC potentials were recorded with a BioSemi system (Amsterdam, Netherlands) using pin-type sintered Ag/AgCl electrodes. Electrode gel was applied to the holders in the headcap and the electrodes were clicked into their holders. In order to avoid artifacts resulting from instabilities caused by the electrolyte-skin-interface [40], recordings were performed at least 60 minutes after electrode attachment. EEG was recorded from frontopolar (Fp1, Fp2), frontal (F3, Fz, F4), central (C3, Cz, C4), parietal (P3, Pz, P4), occipital (O1, Oz, O2), and temporal (T7, T8) locations (according to the 10–20-system), referenced to the CMS-DRL ground and re-referenced off-line to linked mastoids. Recordings were collected from DC to 30 Hz and digitized with a sample rate of 512 Hz. In addition, vertical EOG (above and below the left eye) as well as skin potential (from the palmar side of the left index finger to the extensor side of the forearm) were obtained as control recordings with the same filter settings as for EEG recordings.

### 2.4. Experimental Conditions

#### 2.4.1. Noise

All 48 participants experienced a noise condition and a control condition counterbalanced. In the noise condition, 24 persons experienced white noise and 24 persons experienced irrelevant speech (see Table 1 for details of sound pressure levels [SPLs]). Irrelevant speech was presented by playing backwards a CD of an Austrian comedian (Josef Hader, “Privat”, Audio CD 1995).

**Table 1:** Measures of the SPLs for noise conditions and control condition.

	Leq dB (A)	max dB (A)
*Irrelevant Speech*		
Fast	61.5	71.6
Slow	61.6	66.8
*White Noise*		
Fast	68.7	69.5
Slow	68.8	69.0
*Control*		
Fast	38.2	48.1
Slow	37.6	41.8

#### 2.4.2. Attention Tasks

Two externally directed (*i.e.*, intake) and two internally directed (*i.e.*, rejection) attention tasks [17] with one figural and one verbal task each were developed to investigate the effects of the direction of attention with the consideration of modality. Each task consisted of two parts: the computerized attention task and the examination of task execution, during which the participants were filling in answer sheets in order to verify that the instructions for the attention tasks had been followed.

##### Intake Verbal—“Listening Task”

A memory exercise about a fictitious state in Africa, taken from the *Lern- und Merkfähigkeitstest* [41], a German memory test, was played over a period of 90 seconds, and participants were asked to listen attentively and try to memorize as many facts as possible.

##### Intake Figural—“Recognizing Photographs of Trees”

Participants were presented 10 photographs of various trees, displayed over approximately 75% of the computer screen, over a period of 90 s, with a presentation time of 9 s for each photograph. Participants were instructed to try to remember those photographs.

##### Reject Verbal—“Creating Words”

A syllable taken from the *Verbaler Kreativitäts-Test* [42], a German verbal creativity test, was presented to participants for 90 s and they were asked to create as many words as possible beginning with the respective presented syllable and to try to remember those words. 

##### Reject Figural—“Picture Completion”

Participants were presented four incomplete figures taken from the “Picture Completion” task from the *Torrance Tests of Creative Thinking* [43]. Persons were instructed to complete and memorize those figures for 90 s.

### 2.5. Procedure

Participants were seated in front of a laptop (ACER^©^ Travelmate 291 LCi) with a distance of 80 cm between the person and the laptop screen. The visual angle of the screen measured 20° horizontally and 15° vertically. Prior to the presentation of the attention tasks, persons were informed about the experimental procedure, all attention tasks were explained, and test trials were performed. The experimental procedure started with participants sitting in front of a grey computer screen for one minute with the last 30 s serving as baseline for the first attention task. Each attention task then began with an instructional announcement of the respective task on the computer screen for 20 s, followed by the attention task itself, which lasted 90 s. After the task, examination of task execution took place, lasting 90 s as well. Subsequently, participants sat for 30 s, this epoch served as baseline for the following attention task. In the noise condition, white noise or irrelevant speech was presented continuously for the whole block of the four attention tasks.

### 2.6. Analysis

EEG data were exported with the program BESA (MEGIS Software GmbH, Gräfelfing, Germany) and mean voltage values for each one second window were calculated. DC potential changes were exported for epochs of 230 s, namely 30 s baseline, 20 s of instruction, 90 s of the attention task, and 90 s of the examination of task execution. DC drift artifact correction was performed according to Hennighausen, Heil, and Rösler [44] over a period of 260 s (the epoch of 230 s described above plus 30 s of baseline of the following task) and the first and last 60 s of each 260-s-epoch were used to calculate the slope and eliminate it from the recorded segment in each recording channel. The mean DC potentials from baseline were set to 0 μV and mean values of the attention tasks were calculated for the statistical analysis of the DC potential shifts during the attention tasks.

Statistical analysis was performed with Statistica (Version 7, StatSoft Inc., Tulsa, OK, USA) and an alpha level of *p* = 0.05 was applied for all statistical tests. MANOVA was performed for DC potentials with the factors location (Fp1, Fp2, F3, Fz, F4, C3, Cz, C4, P3, Pz, P4, T7, T8, O1, Oz, O2), direction of attention (intake *vs.* rejection), modality (figural *vs.* verbal), and noise condition (no-noise *vs.* noise) as within factors and with noise type (irrelevant speech *vs.* white noise) as the between groups factor. For EOG and skin potential, the same analyses as for DC potentials were performed, however, without the factor location. Three persons had to be excluded from analyses because of recording artifacts.

## 3. Results

**Figure 1:**
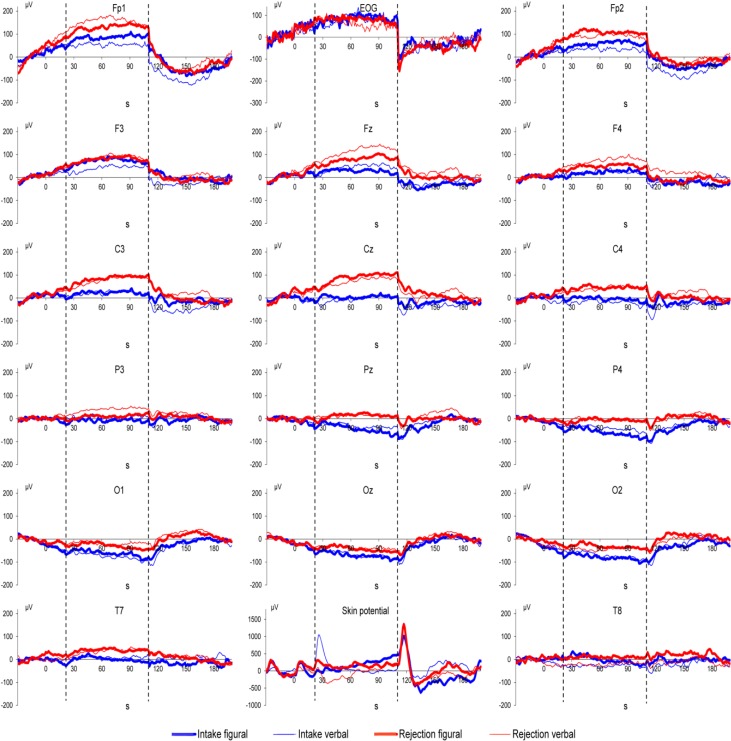
No-noise condition: Grand means of brain DC potentials (positivity upward) as well as vertical EOG and skin potential for the four attention task conditions. The timeline indicates when instruction (seconds 1–20), attention tasks (seconds 21–110; between the vertical broken lines), and examination of task execution (seconds 111–200) took place.

**Figure 2:**
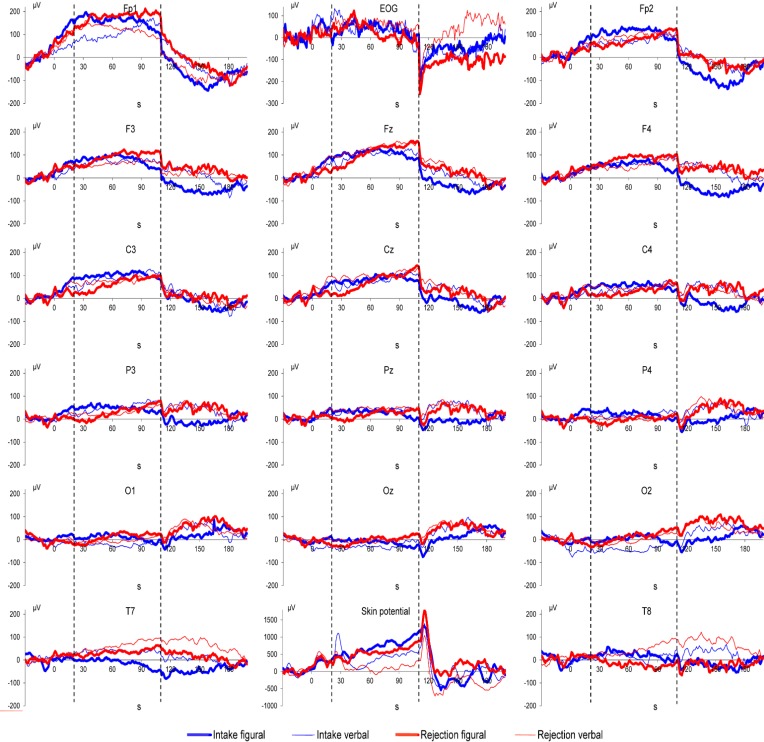
Irrelevant speech condition: Grand means of brain DC potentials (positivity upward) as well as vertical EOG and skin potential for the four attention task conditions. The timeline indicates when instruction (seconds 1–20), attention tasks (seconds 21–110; between the vertical broken lines), and examination of task execution (seconds 111–200) took place.

**Figure 3:**
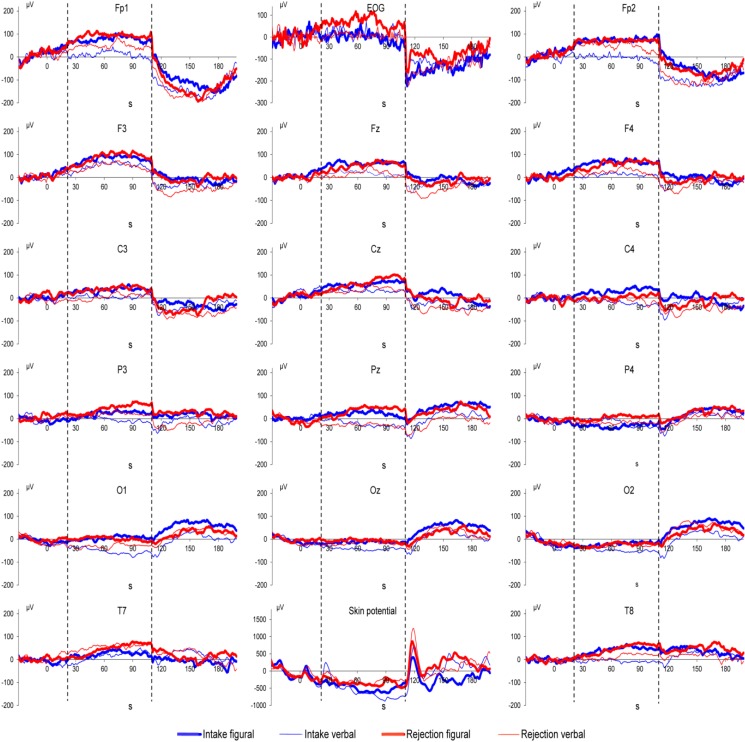
White noise-condition: Grand means of brain DC potentials (positivity upward) as well as vertical EOG and skin potential for the four attention task conditions. The timeline indicates when instructions (seconds 1–20), attention tasks (seconds 21–110; between the vertical broken lines), and examination of task execution (seconds 111–200) took place.

**Figure 4:**
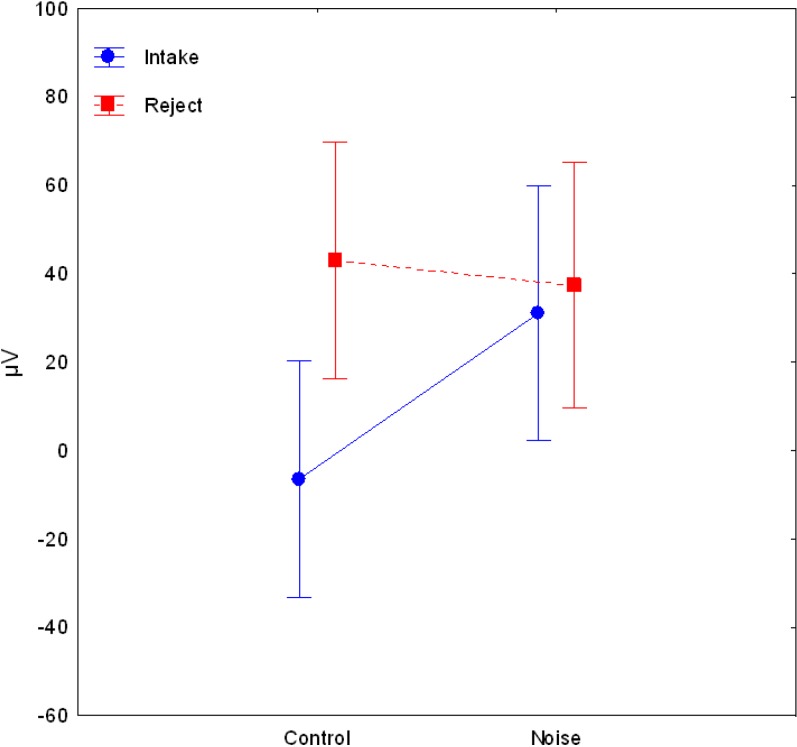
Mean values (±95% CI) of pooled brain DC potentials for intake and rejection tasks in control condition and noise condition.

Grand means of DC potential changes as well as of vertical EOG and of skin potential are displayed in Figure 1, Figure 2 and Figure 3. MANOVA results showed a main effect of Location (*F*(15,29) = 8.41, *p* < 0.001) and Direction of Attention (*F*(1,43) = 4.51, *p* = 0.039). MANOVA also showed an interaction between Noise and Location (*F*(15,29) = 2.07, *p* = 0.044), indicating more positive DC potential changes in the noise condition compared to the control condition (as can be seen in Figure 1, Figure 2 and Figure 3). In detail, significant effects (as indicated by LSD test) were observed at electrodes Cz, T8, P3, Pz, P4, O1, Oz, and O2. Furthermore, the interaction of Noise × Direction of Attention (*F*(1,43) = 4.46, *p* = 0.040) indicates that in the control condition rejection tasks are associated with more positive DC potential changes compared to intake tasks (Figure 1 and Figure 4), in the noise condition, however, DC shifts of intake tasks became more positive and reached the level of rejection tasks (Figure 1, Figure 2, Figure 3 and Figure 4). A trend for an effect of modality was observed in the interaction of Location × Modality (*F*(15,29) = 1.82, *p* = 0.080), with confidence intervals indicating more positive DC potentials for verbal tasks compared to figural tasks at electrode P3. No other effects or interactions were statistically significant. Analysis of EOG revealed one statistically significant effect for Noise (*F*(1,43) = 7.73, *p* = 0.008), indicating lower values in the noise condition compared to the control condition (Mean ± SD: 30.09 ± 13.90 in the noise condition *vs.* 77.80 ± 14.08 in the control condition). No such effect was observed in frontopolar locations (*F*(1,43) < 1, *p* = n.s.) which suggests that confounding of EEG recordings by EOG is unlikely. For skin potential, the only significant effect was a main effect of Noise Type (*F*(1,43) = 13.79, *p* < 0.001) with higher values in the irrelevant speech group compared to the white noise group (Mean ± SD: 601.33 ± 197.04 for irrelevant speech *vs.* −422.18 ± 192.71 for white noise). No such effect was observed for pooled EEG recordings ((*F*(1,43) = 1.24, *p* = 0.27); Mean ± SD: 35.20 ± 11.42 for irrelevant speech *vs.* 17.41 ± 11.17 for white noise).

## 4. Discussion

Results indicate different brain DC potentials for intake and rejection tasks in the control condition. It could be shown that without noise, tasks requiring internally directed attention showed more positive DC shifts compared to externally directed attention tasks. Under noise conditions, however, this difference disappeared since environmental rejection tasks as well as intake tasks were associated with positive DC potential shifts. Analyses of control recordings (EOG, skin potential) revealed different patterns compared to effects of brain DC potentials, which makes confounding of the obtained results of brain DC potentials unlikely. However, possible confounding of the results due to the lack of control for blood brain barrier pCO_2_ changes cannot be completely ruled out.

It is assumed that, as an expression of selective attention, information that is not relevant to a task will be inhibited in order to facilitate the processing of relevant information [13]. In the present study, DC shifts towards positivity are suggested to reflect this inhibition (*i.e.*, rejection) process. This view is supported by studies investigating DC potential shifts both from scalp recordings [15,31] as well as from single cell recordings [32,35], which suggest surface positive DC potential changes to reflect cortical inhibition.

The effect that direction of attention as intake *vs.* rejection is associated with different/altered electrocortical activity was also found analyzing alpha activity [19,20]. Results of this study suggest that when trying to filter non-relevant environmental information, participants performing intake tasks show positive DC potential shifts comparable to those of participants performing rejection tasks. This could be interpreted as an additional cognitive load caused by acoustic background noise, which leads to altered information processing of intake tasks as an expression of an inhibition of environmental noise. This interpretation is supported by an investigation on the effects of low intensity background noise on DC potentials of an attention task, where positive shifts were interpreted as a “perceptual defense” response to noise [9]. Furthermore, positive shifts recorded directly from the brain in rat experiments were discussed to represent “protective inhibition” [45] of a “death cry” of another rat being snapped by a snake. The view that top-down processes as expressed by the direction of attention alter cortical activity by means of facilitation/inhibition according to the task on hand is supported by Ghatan *et al.* [46] investigating effects of irrelevant speech by fMRI, where noise-dependent changes in cerebral blood flow were interpreted as a sign of “inhibitory modulation” of non-task-relevant information. A recent animal study (three-spined stickleback, *Gasterosteus aculeatus*) [47] suggests that exposure to acoustic noise results in “decreased foraging efficiency” by interfering with attention processes, our study could be interpreted as a possible display of the underlying mechanism of such an “attention shift”.

Thus one can speculate that the attention shift caused by environmental noise is the underlying process for the observed harmful effects on learning, memory, and performance. There is good empirical evidence that environmental noise diminishes cognitive learning and memory performance in school classes [48,49,50,51,52,53]. Such an effect would be predicted by this study, because the outcome of our investigation displays a change of the brain DC potential towards positivity in intake tasks during noise. It is well established that positive brain DC potentials, as an expression of cortical inhibition, are associated with lowered performance in cognition and motor behavior [54,55]. Moreover, in intake tasks during noise an additional mental process seems to be active, namely an environmental noise rejection process, representing an additional mental task load. This then may be associated with extra mental effort (as supported by the observation of the coincidence of positive DC shifts with task load [56,57]) caused by task switching as recently suggested in a theoretical approach [58]. The higher mental effort for learning during environmental background noise was recently supported by the analysis of spontaneous skin conductance fluctuations as an indication of the activity of the sympathetic nervous system [10]. Thus there is converging evidence that environmental noise affects cognitive performance and memory processes by directing attention on environmental events. That means that an additional mental process is necessary, namely filtering out non-relevant environmental information, which in turn leads to a kind of a "dual task paradigm" if one is already engaged in a cognitive task, associated with less performance and higher mental effort. From dual task experiments it is well established that due to limitations in resources [59], the performance of cognitive modules which are engaged in two tasks of the same modality at the same time—e.g., perception of auditive information and filtering out auditive noise—shows reduced performance and needs higher mental effort compared to a single task.

A trend for an effect of modality could be observed by more positive DC potential shifts for verbal tasks compared to figural tasks at location P3. This could be interpreted as an additional challenge for the left hemisphere (*i.e.*, the language-dominant hemisphere in right-handed persons) during verbal tasks. This trend, however, was independent of the presence of background noise. A limitation of the study can be seen by not having recorded respiration rate to rule out that the interaction of direction of attention with noise could be affected by changes in pCO_2_ [24,25,26]. However, according to Vuopio *et al.* [25], pCO_2_-induced DC changes occur in the range of minutes, whereas in our study, DC shifts occur within seconds. Furthermore, this would also imply that the respiration change only appeared for the intake tasks under noise. This argument also applies to the view that the observed effects may be caused by stress during noise. Additionally, one could argue that the figural rejection task requires both internally and externally directed attention. However, the observed noise effect appeared for figural as well as verbal tasks. Moreover, in the no-noise condition, intake and rejection tasks could be differentiated by means of DC potentials in both the figural and the verbal task, suggesting some evidence that the tasks differed in the orientation of attention.

Practical considerations can be drawn for brain research and for public health. The present study demonstrated that noise effects modulate brain activity by mechanisms of attention, and might therefore give some theoretical background on a fundamental principle responsible for widely observed noise effects. This includes consequences for brain research methods like fMRI; as that can be associated with a considerable noisy environment, one should take into account that under such environmental noise condition altered attention mechanisms may be evoked and would bias the results as also suggested by Hommel *et al.* [60].

Practical consequences for public health are also obvious. (1) The attenuation and/or avoidance of environmental noise not only for health and well-being but also to avoid detrimental effects on cognitive performance and to avoid additional mental effort during cognitive tasks, in particular (2) in learning, educational, and performance environments like schools and universities [61]; (3) Furthermore, especially susceptible persons who suffer from ADHD [62], dyslexia [63], or hearing impairment [64] as well as elderly persons [65,66] are in particular challenged by noise.

## 5. Conclusions

Without noise, intake and rejections tasks can be differentiated by brain DC potentials. However, this effect disappears under task conditions of environmental noise. This may indicate an attention shift in that way, that noise challenges selective attention processes by switching to an environmental information processing and noise rejection mode. Practical considerations include that fMRI studies could be biased by noise. Furthermore, regarding public health, noise in learning and performance environments (schools, universities, *etc.*) should be avoided/attenuated, in particular considering susceptible persons who suffer from ADHD, dyslexia, or hearing impairment and elderly persons.
